# Self-Monitoring of Urinary Hormones in Combination with Telemedicine — a Timely Review and Opinion Piece in Medically Assisted Reproduction

**DOI:** 10.1007/s43032-021-00754-5

**Published:** 2021-11-15

**Authors:** Roger J. Hart, Thomas D’Hooghe, Eline A. F. Dancet, Ramón Aurell, Bruno Lunenfeld, Raoul Orvieto, Antonio Pellicer, Nikolaos P. Polyzos, Wenjing Zheng

**Affiliations:** 1 Division of Obstetrics and Gynaecology, The University of Western Australia & Fertility Specialists of Western Australia, Perth, WA Australia; 2 Department of Development and Regeneration, KU Leuven, Leuven, Belgium; 3 Global Medical Affairs Fertility, R&D Healthcare, the healthcare business of Merck KGaA, Frankfurter Str. 250, 64293 Darmstadt, Germany; 4 Department of Obstetrics and Gynecology, Yale University, New Haven, CT USA; 5 Leuven University Fertility Centre, University Hospitals Leuven, Leuven, Belgium; 6 IVF Unit, Fertility Campus Hospital Quirónsalud Barcelona, Barcelona, Spain; 7 Faculty of Life Sciences, Bar-Ilan University, Ramat Gan, Israel; 8 Infertility and IVF Unit, Department of Obstetrics and Gynecology, Chaim Sheba Medical Center (Tel Hashomer), Ramat Gan, Israel; 9 Sackler School of Medicine, Tel Aviv University, Tel Aviv, Israel; 10 The Tarnesby-Tarnowski Chair for Family Planning and Fertility Regulation, Sackler Faculty of Medicine, Tel-Aviv University, Tel Aviv, Israel; 11 Instituto de Investigación Sanitaria La Fe, Valencia, Spain; 12 Department of Obstetrics, Gynecology and Reproductive Medicine, Dexeus Mujer, Dexeus University Hospital, Barcelona, Spain; 13 Faculty of Medicine and Health Sciences, Ghent University, Ghent, Belgium

**Keywords:** Home-based monitoring, In vitro fertilization (IVF), Medically assisted reproduction (MAR), Self-monitoring, Telemedicine, Remote urine-based hormonal monitoring

## Abstract

Cycle monitoring via ultrasound and serum-based hormonal assays during medically assisted reproduction (MAR) can provide information on ovarian response and assist in optimizing treatment strategies in addition to reducing complications such as ovarian hyperstimulation syndrome (OHSS). Two surveys conducted in 2019 and 2020, including overall 24 fertility specialists from Europe, Asia and Latin America, confirmed that the majority of fertility practitioners routinely conduct hormone monitoring during MAR. However, blood tests may cause inconvenience to patients. The reported drawbacks of blood tests identified by the survey included the validity of results from different service providers, long waiting times and discomfort to patients due to travelling to clinics for tests and repeated venepunctures. Historically, urine-based assays were used by fertility specialists in clinics but were subsequently replaced by more practical and automated serum-based assays. A remote urine-based hormonal assay could be an alternative to current serum-based testing at clinics, reducing the inconvenience of blood tests and the frequency of appointments, waiting times and patient burden. Here we provide an overview of the current standard of care for cycle monitoring and review the literature to assess the correlation between urine-based hormonal assays and serum-based hormonal assays during MAR. In addition, in this review, we discuss the evidence supporting the introduction of remote urine-based hormonal monitoring as part of a novel digital health solution that includes remote ultrasound and tele-counselling to link clinics and patients at home.

**Supplementary Information:**

The online version contains supplementary material available at 10.1007/s43032-021-00754-5.

## Introduction

Cycle monitoring has been established as a standard of care of medically assisted reproduction (MAR) and has been used for nearly 60 years to evaluate ovarian response and, more importantly, to help shape individualized treatment plans [1, 2]. The clinical value of cycle monitoring is evident in choosing a stimulation protocol and guiding dose adjustments. During controlled ovarian stimulation (COS), cycle monitoring can assist in defining the ideal timing for ovulation triggering, with the intent of obtaining an optimal number of good quality eggs while reducing complications such as ovarian hyperstimulation syndrome (OHSS).

Cycle monitoring usually involves a transvaginal ultrasound to assess developing follicle size and number, and serum hormonal testing to determine hormone concentrations at different stages of COS. However, the addition of serum hormonal monitoring to ultrasound for cycle monitoring has been widely debated in recent years, due to the lack of evidence of added value in terms of fertility outcomes when compared to cycles monitored with ultrasound alone [3]. Nevertheless, today, hormonal monitoring during COS using serum-based assays remains a common practice in clinics worldwide [4–6].

Serum assays are not the only way to determine hormone levels. Indeed, historically, hormone levels were routinely measured in the clinic by urinary hormone assays that required sophisticated handling techniques [7]. Over the years, the advent of serum assays, with a well-established reference range and the ability for automation, essentially replaced the use of urinary assays. However, remote urine-based hormonal testing could be an alternative method of monitoring. This monitoring method could potentially reduce the frequency of venepuncture, thereby limiting the potential physical (i.e. injection-related pain) and emotional (i.e. injection anxiety) burden that may be experienced by patients [8, 9]; indeed, the number of injections has been shown to affect women’s choice of fertility medication [10]. Other advantages include not requiring costly skilled personnel for blood collection, and a reduction of the frequency and duration of clinic visits (e.g. time in the waiting room and travelling time), which has been reported to have a considerable negative effect on patient experience during fertility treatment [11, 12].

The ongoing coronavirus disease 2019 (COVID-19) pandemic has put MAR treatment on hold for many patients to avoid overburdening healthcare systems. This discontinuation of reproductive care, for all but urgent cases, was advised at the beginning of the pandemic by both the European Society of Human Reproduction and Embryology (ESHRE) and the American Society for Reproductive Medicine (ASRM) [13]. A recent study showed that discontinuing IVF in the USA for just 1 month results in 369 fewer women having a live birth, mainly due to women ageing during the shutdown of fertility clinics [14, 15]. Evidence also suggests that the pandemic has a severe psychological impact on infertile patients and is a major source of stress and anxiety [16, 17]. Given the impact of infertility on people’s quality of life and well-being, the World Health Organization (WHO) and major societies for reproductive medicine have expressed the importance of sustaining reproductive care in light of the pandemic, defining infertility as both a disease and disability [13, 18, 19]. Thus, the pandemic has led to the re-evaluation of clinical practices, demonstrating the potential value of remote testing to limit the frequency and duration of clinic visits.

Notwithstanding its potential value, particularly in the current and post-COVID-19 eras, remote urine-based hormonal testing needs to be thoroughly investigated before it can be implemented in clinical practice. Here, we describe current cycle monitoring, review studies on urine- and serum-based hormonal assays and reflect on the value for clinical practice of combining the possibility of self-operated home sonography with state-of-the-art remote urine-based testing.

## Current Standard of Care

### Use of Ultrasound and Serum-Based Hormone Monitoring by Fertility Practitioners

To assess the use of ultrasound and hormonal monitoring during COS, we conducted two online surveys in December 2019 and December 2020 including 7 and 17 fertility specialists, respectively, from Europe, Asia and Latin America. The surveys comprised questions assessing the frequency and role of hormonal monitoring, hormones tested and drawbacks of blood tests, with the specialists providing their answers as short free text or selecting from multiple choice options.

The results from the surveys are summarized in Supplementary Table 1 and Supplementary Table 2. A majority of the participants (7/7 and 15/17 of respondents) indicated that they routinely check serum hormonal levels during ART treatment. Overall, 7/7 and 14/17 of specialists reported that they believe that regular hormonal tests (as part of combination monitoring with ultrasonography) have additional value when guiding treatment decisions, in terms of improved pregnancy and safety outcomes. According to the results of our second survey in 2020, E2, P4 and LH were the most common routinely measured serum markers during MAR in clinical practice (Supplementary Table 2).

Based on the results from 2019 survey, all specialists agreed that E2 levels are useful when guiding dose adjustments, 5 out of 7 agreed that they see E2 levels as an indicator of OHSS and 5 out of 7 agreed that the peak of E2 level is a good indicator of the optimal time for ovulation triggering (Supplementary Table 1). Similarly, during the second (2020) survey, 13 out of 17 specialists considered that E2 measurement is useful when adjusting gonadotropin dose. In addition, the majority (15 out of 17) of specialists also thought that E2 is an important marker for OHSS risk. The peak of E2 levels was viewed as a useful predictor of the optimal timing of ovulation triggering by 8 out 17 specialists (Supplementary Table 2). Besides hormonal monitoring, all 17 specialists from the second survey confirmed that they also use ultrasound assessment and reported that they consider the combination of both to be the optimal approach for cycle monitoring.

Apart from E2, P4 was also recognized by all specialists (7/7) in the first survey in 2019 as an indicator to assist decision making for embryo transfer strategy (fresh vs frozen-warmed embryo transfer) (Supplementary Table 1).

### Overview of the Current Guidelines for Cycle Monitoring during MAR

The two surveys clearly showed that although ultrasound is the mainstay of monitoring, the addition of hormonal monitoring is still perceived as valuable to clinical practice by fertility practitioners. We then reviewed relevant national and international society guidelines and recommendations on the current standard of care for hormonal monitoring in different scenarios in the context of MAR.

The prediction of ovarian response prior to treatment (based on the assessment of individual patient characteristics and hormonal profile at baseline) is a common clinical practice during MAR. According to the 2019 ESHRE guidelines on COS for IVF/intra-cytoplasmic sperm injection (ICSI), serum markers such as anti-Müllerian hormone (AMH) and follicle-stimulating hormone (FSH), and patient characteristics such as age and antral follicle count (AFC) are good predictors of ovarian response and should be used to guide clinicians in selecting an optimal COS protocol [20]. In addition, the ASRM suggests that serum E2 could also be used to predict ovarian reserve when interpreted in combination with basal serum FSH, on the basis that basal E2 values of > 60‒80 pg/mL have a suppressive effect on FSH levels and, therefore, could be indicative of decreased ovarian reserve [21, 22].

Individualized treatment decisions during COS, including selection of appropriate protocols (e.g. gonadotropin-releasing hormone [GnRH] agonist or antagonist), gonadotropin type and dose, type of trigger of final oocyte maturation and type and duration of luteal phase support, are essential to ensure that the patient’s response to treatment is optimized with respect to efficacy and safety [2, 23–25]. To this end, starting dose selection according to predicted ovarian response prior to stimulation, and dose adjustments during treatment cycle are frequently performed in fertility clinics [26, 27]. Assessment of hormonal profile plays an important role in almost all aspects of individualized fertility treatment, but particularly in guiding intra-cycle dose adjustments, as these are directly based on ultrasound assessment of follicular development and monitoring of serum hormones [20]. Individualizing gonadotropin dose according to patient response is essential in mitigating the risk of inadequate ovarian response to stimulation and subsequent cycle cancellations in poor responders [28, 29], and in decreasing the risk of OHSS in hyper responders [30].

Nevertheless, there is a lack of uniformity in the clinical approaches used for cycle monitoring during COS in fertility clinics worldwide, for which conflicting data may play a major role. In a Cochrane meta-analysis by Kwan et al. in 2014 including six randomized controlled trials, the addition of serum E2 monitoring to ultrasound during MAR did not appear to increase the probability of pregnancy or the number of oocytes retrieved, nor did it decrease the probability of detecting OHSS [3]. In the referred study, a GnRH agonist protocol was applied to more than 70% of the patients analyzed, leaving it unclear as to whether the aforementioned conclusion is only applicable to GnRH agonist cycles [3]. Based on this evidence, ESHRE guidelines state that the addition of basal serum E2, P4 and luteinizing hormone (LH) monitoring to the conventional ultrasound assessments during COS is ‘probably not recommended’ [20]. However, these recommendations are ‘conditional’, meaning that the quality of evidence underpinning these conclusions was considered to be low overall and different choices will be appropriate for different patients; therefore, shared-decision making is recommended [31].

In contrast, the ASRM 2016 guideline on the prevention and treatment of moderate and severe OHSS, found ‘fair evidence’ that serum E2 concentrations, among other factors including elevated AMH levels, multifollicular development and a high number of oocytes retrieved, are associated with an increased risk of OHSS. Specifically, an E2 cut-off value of 3500 pg/mL measured around the day of ovulation triggering during COS was identified as predictive of an increased risk of OHSS [32]. In a review of evidence aimed at informing the WHO guideline development group on the global management of COS, the authors concluded that combined ultrasound and E2 monitoring should be retained at least in women with a high risk of OHSS [32, 33].

The combination of hormonal data with ultrasound monitoring has been used to determine the time of ovulation triggering in COS cycles, and has been recognised as ‘good practice’ by the ESHRE guidelines: “The decision on timing of triggering in relation to follicle size is multi-factorial, taking into account the size of the growing follicle cohort, the hormonal data on the day of pursued trigger, duration of stimulation, patient burden, financial costs, experience of previous cycles and organizational factors for the centre.” [20]

The Practice Committees of the ASRM and the Society for Reproductive Endocrinology and Infertility (SREI) support the opinion that the safety and efficacy of ovulation induction in infertile women who are anovulatory depend on both careful monitoring with ultrasound and assessment of hormone concentrations, as their combined use accurately reflects the response to treatment, thereby enabling informed decisions regarding treatment management strategies to be made [34]. During ovulation induction with clomiphene citrate, monitoring usually includes ultrasound and measurement of serum E2 and P4 levels to indicate whether ovulation has occurred [35, 36]. Step-up and step-down gonadotropin protocols can also be used for ovulation induction and, with both of these protocols, serial ultrasound and serum E2 measurements are necessary to adjust the dose of gonadotropins and consequently decrease the risk of multiple gestation and OHSS [37]. The monitoring of urinary LH has been used in clinical practice to detect a peak in hormone levels 5–12 days after completion of ovulation induction with clomiphene citrate; this can then be used to determine ovulation and the subsequent interval of peak fertility to plan the timing of intercourse or intrauterine insemination [35].

Premature luteinisation in ART as evidenced by P4 elevation has been a controversial topic for decades [38]. As recommended by Kaponis et al., follicular-phase progesterone rise (FPPR) may be a more accurate term since it is not always dependent on LH and may happen before the day of hCG. FPPR may have detrimental impact on both endometrium and oocyte quality [38, 39]. The survey in 2019 confirmed that fertility practitioners opt to use frozen-warmed embryo transfer cycles to avoid the potential negative impact of progesterone elevation. However, there is no national or international guideline for prediction of cycle prognosis or decision making for elective frozen-warmed embryo transfer based on serum P4 levels during ovarian stimulation.

### Patient Perspective of Cycle Monitoring

Cycle monitoring, specifically hormonal assessment via blood tests, may be burdensome for women undergoing treatment for infertility. As part of the Millennium Cohort Study, a questionnaire-based evaluation of 230 women successful in achieving a pregnancy was conducted to assess patient experience of fertility treatment and care [9]. Women described blood tests as one of the many frustrating obligations during infertility treatment [9]. In addition, in a study of 276 IVF patients completing the FertiMed questionnaire evaluating feedback on medication characteristics, it was shown that anxiety related to subcutaneous injections was negatively affecting patients [8]. As such, it is evident that some patients fear injections and that limiting the number of venepunctures could reduce anxiety and improve overall patient experience during treatment.

Waiting time for different assessments and procedures, as well as treatment delays, are some of the negative aspects of infertility treatment frequently reported by patients [11]. Cycle monitoring at the clinic, including hormonal tests and/or ultrasound, is likely to be associated with increased time in the waiting room thus interfering with patients’ daily lives. A study by Brod and Fennema, using the Controlled Ovarian Stimulation Impact Measure (COSI) in 267 patients undergoing treatment for infertility, showed that women with fewer clinic visits had superior patient-reported outcomes with respect to the overall COSI score and to the specific domain of interference with daily life [12]. Development of remote urine-based hormonal testing could potentially help eliminate some of the burdensome aspects related to waiting times and daily life interference, and help patients feel more in control of their treatment [9].

Despite feedback regarding the negative effect of clinic waiting times and frequent visits, the importance of doctor–patient interaction and face-to-face support from clinic staff should not be dismissed. Patients have described fertility treatment as a physically and emotionally painful and stressful process, followed by the feelings of depersonalization, and absence of dignity and respect [9]. However, the value of good communication and relationships with physicians and clinic staff has also been recognised [9, 11, 40–42]. Interestingly, evidence suggests that weaknesses from system factors of patient-centred care (such as frequent clinic visits, long waiting times and physical discomfort) can be compensated for by positive human factors (such as relationship and communication with clinic staff) [42].

In general, there is a lack of research on the aspects specific to cycle monitoring (blood testing and ultrasound) that patients find important and/or burdensome. Waiting times and daily life interference are negatively associated with patient experience, while more effective doctor–patient interaction is appreciated by patients. There is a need for further studies to identify these aspects, attenuate treatment-related stress and facilitate patient-centred treatment.

## Urine-Based Hormonal Assays

A comprehensive literature review was performed to assess the validity, clinical utility and potential application of urine-based assays for the monitoring of reproductive hormones (Tables 1 and 2). The PubMed search engine was used to search the Medline database between 1950 and 2020.

**Table 1:** Summary of systematic literature search criteria

Database(s) searched	Medline (via PubMed)
Cut-off date for publication	1950–2020
Key words/search terms	(MeSH-) search terms related to cycle monitoring (e.g. fertility monitoring, controlled ovarian stimulation, ovulation confirmation) and urinary hormonal assays (e.g. estrone-3-glucuronide or E1-3G, pregnanediol-3-glucuronide or PdG)
Screening criteria for inclusion^a^	Studies reporting on the correlation between serum reproductive hormones and urinary hormone metabolites in gonadotropin stimulated or natural cycles

Results were automatically filtered to include studies in humans and publications in English, and duplicates were removed

^a^Based on titles and abstracts

**Table 2:** Summary of systematic literature search result

Reference	Patient population	Indication
Hobkirk et al., 1974 [59]	4 Non-pregnant women	Urinary assay development
Wright et al., 1978 [51]	N/A	Urinary assay development
Baker et al., 1979 [55]	11 Women with normal cycles	Urinary assay development
Branch et al., 1982 [56]	6 Women (aged 22–28 years) with normal cycles	Urinary assay development
Frenkel et al., 1985 [54]	28 Infertile women	Urinary assay development
Alessio et al., 1985 [67]	Group 1: 271 from the general population, group 2: 105 exposed to cadmium, group 3: 16 men	Urinary assay development
Miller et al., 2004 [66]	30 Women	Urinary assay development
Sawant et al., 2018 [68]	120 Healthy individuals	Urinary assay development
Newman et al., 2019 [49]	4 Premenopausal and 8 Postmenopausal women	Urinary assay development
Denari et al., 1981 [53]	24 Women with normal cycles	Application of urinary assay
MacLean et al., 1981 [71]	12 Women (22 cycles)	Application of urinary assay
Thornton et al., 1990 [65]	24 Women (57 cycles)	Application of urinary assay
Blackwell et al., 2018 [49]	N/A	Application of urinary assay
Lessing et al., 1987 [58]	31 Patients with mean age 32 (24–40), from D3 with 225 IU hMG for ovulation induction	Stimulation cycle
Catalan et al., 1989 [52]	14 Women (aged 27 to 36 years), ovulation induction CC + 75 IU hMG)	Stimulation cycle
Rapi et al., 1992 [47]	24 Patients (31 cycles), GnRH-a long protocol, 225 IU hMG	Stimulation cycle
Alper et al., 1994 [60]	25 Patients (age 29–39), GnRH – a short protocol, 150–300 IU hMG (3 to 6 samples per patient)	Stimulation cycle
Borth et al., 1957 [44]	5 Women (aged 22–34)	Natural cycle
Stanczyk et al., 1980 [57]	7 Women (aged 24–40)	Natural cycle
Pazzagli et al., 1987 [64]	14 Women (aged 21–36)	Natural cycle
Catalan et al., 1989 [52]	10 Healthy women (aged 23–33)	Natural cycle
Munro et al., 1991 [61]	10 Healthy women (aged 23–40)	Natural cycle
Kesner et al., 1994 [61]	13 Normal and 6 atypical menstrual cycles	Natural cycle
O ‘Connor et al., 2003 [63]	30 Women with 34 paired days	Natural cycle
Roos et al., 2015 [45]	40 Women (aged 18–40)	Natural cycle

Results were automatically filtered to include studies in humans and publications in English, and duplicates were removed. Finally, 13 publications on urinary hormone assay development and application, and 12 publications on correlation between serum and urinary hormone assay in natural cycles and gonadotropin stimulated cycles were included for analysis

### Validation of Urine-Based Hormonal Assays for Monitoring Reproductive Hormones

Serum-based hormonal assays provide direct measures of hormones circulating in the blood at a specific time point. Hormone concentrations in the blood can vary throughout a 24-h period. Diurnal variation has been observed for several reproductive hormones, the rhythm of which is dependent on the menstrual cycle [43, 44], which in turn is subject to considerable intra- and inter-individual variation [45]. Serum hormone measurement may therefore provide variable results, not only dependent on the day of blood collection but also on the time of the day. For example, Filicori and colleagues have reported that during the luteal phase, serum progesterone levels can fluctuate from as low as 2.3 ng/ml to peaks of 40.1 ng/ml throughout a 24-h period [46]. Indeed, the inter- and intra-cycle variations in hormone concentrations and the effect of circadian rhythm were identified as one of the perceived drawbacks of serum hormone monitoring by some of the respondents to our survey (Supplementary Table 1). In contrast, a urine-based hormonal assay reflects average hormone levels over time of excretion into the bladder, typically an 8–10-h time period [47]. Indeed, a recent study has demonstrated that measuring of urinary hormone levels at four time points accurately represents results from a complete 24-h period [48].

Being direct products of the ovary, changes in the levels of E2 and P4 and their metabolites excreted in the urine are directly related to the underlying ovarian physiology [49]. Estrone-3-glucuronide (E1-3G) and pregnanediol-3-glucuronide (PdG) are recognised by the WHO as the principal metabolites of E2 and P4 in urine, respectively [50]. It has been demonstrated that E2 metabolites usually reach the urine 12‒24 h after free E2 appears in blood [51]; therefore, it is best to measure E1-3G in the early morning urine sample, as it reflects overnight levels of E2 metabolites in urine. Indeed, the use of early morning urine samples to measure E1-3G was previously suggested to be as informative as using urine samples collected over 24 h [52]. As such, the first morning voiding is generally considered the sample of choice to accurately determine both E1-3G and PdG, due to its ease of collection and its high correlation with plasma E2 and P4 levels [53, 54].

E1-3G has also been demonstrated to be the most useful E2 metabolite to measure in urine as it demonstrates a high mid-cycle peak-to-baseline ratio [55, 56], a low degree of variation between individuals [55] and a good correlation with serum E2 [56–59]. Furthermore, a relatively good correlation between serum E2 and urinary E1-3G has been observed in both stimulated [47, 52, 58, 60] and natural cycles [45, 52, 57, 61–64].

A number of urine-based immunoassays have also been assessed for monitoring urinary PdG. While there is a lack of studies assessing the use of these methods in gonadotropin-stimulated cycles, some studies have been performed in women with natural cycles. Stanczyk et al. (1980) measured immunoreactive metabolites of E2 and P4 directly in diluted 24-h urine samples from seven fertile women with regular ovulation, correlating these with the corresponding serum measurements. Analysis showed that the urinary excretion of PdG increased parallel to serum P4 levels and the authors concluded that the measurements of PdG are useful for the detection of ovulation [57].

Urinary E1-3G and PdG can be quantified in several ways: based on the excretion time (i.e. measuring in nmol/24 h, nmol/h, etc.) [47, 55, 64, 65], by measuring creatinine-corrected concentration [55, 57, 59, 66] or by measuring absolute concentration [52, 54]. Whether correction for creatinine concentration is necessary or not for monitoring metabolites of ovarian hormones in urine remains unclear; indeed, creatinine excretion itself is not consistent and depends on gender, age, activity and diet [45, 66–68]. Pazzagli et al. (1987) investigated the potential benefits of using urinary creatinine excretion or overnight voiding volume of urine to correct for day-to-day variations in diuresis [64]. Both approaches increased the coefficients of variance of PdG between subjects with respect to absolute hormone concentration measurements (62.6% when correcting for overnight voiding volume, 53.5% when correcting for creatinine excretion, compared to 45.0% uncorrected) and within subjects (38.8% when correcting for overnight voiding volume, 30.5% when correcting for creatinine excretion, compared to 22.0% uncorrected).

Many assays have been shown to be appropriate for urinary hormone testing and are generally considered to be accurate and reliable in comparison to serum tests. Each assay has associated advantages and disadvantages, and are commonly used for monitoring E1-3G and PdG (Table 3). Recent developments have enabled home testing of urinary E1-3G and LH by patients via point of care (POC) devices [69].

**Table 3:** Summary of advantages and disadvantages of assays used for urinary hormone monitoring

Assay	Hormones tested	Advantages and disadvantages
Radioimmunoassay (RIA)	E1-3G	• Excellent correlation between E1-3G and serum E2 (optimal urine dilution of 1:200) [52] • Related hazards and drawbacks of handling radioactive material
Chemiluminescence immunoassay (CIA)	E1-3G PdG	• Provide the stability and sensitivity to detect E1-3G in urine samples, is not significantly affected by background interference, and can be applied to diluted urine without prior purification, with results obtained within 2.5 h [58] • High correlation between urine CIA and serum RIA findings (Pearson’s correlation coefficient 0.92; *P* < 0.0001) although discrepancies were observed for 23% of patients due to hormone pulsality in blood rather than the urine samples [47] • Successfully used to monitor urinary PdG in normally menstruating healthy women [64]
Enzyme immunoassay	Estrone E1C PdG	• Excellent intra-individual correlation between urinary estrone and serum E2, and urinary E1C and serum E2 [60, 62] • Effect of gonadotropins on E2 metabolism may impact ability of urinary E1C to predict serum E2 at higher values [60] • Shown to be accurate a reliable for monitoring of urinary PdG and E1C [62, 63]
Fluoroimmunoassay	E1-3G PdG	• Validated use for measuring E1-3G and PdG [61] • Correlation between urinary hormone profiles and serum profiles with a 1–2 day delay in urine profiles due to steroid metabolism [45]

### Comparison of Urinary and Salivary Hormone Tests

Salivary tests, like urine tests, are a non-invasive option for home hormone monitoring [70]. Despite the similar benefits of these methods, salivary assays may be less convenient to patients, since the samples must be sent to a laboratory as the assay (enzyme-linked immunosorbent assay) cannot be done at home [5, 70]. This then adds to the time that clinicians and patients will have to wait for test results to become available. Like urinary tests, salivary E2 correlates well with serum E2; however, salivary P4 correlates poorly with serum P4, decreasing the potential applications of this measurement [5].

### Areas of Application of Urine-Based Hormonal Assays in the Context of MAR

There are a number of ways in which urine-based hormonal assays could be applied in the context of the diagnosis of female infertility and treatment with MAR.

With respect to ovulatory function, specific values related to the PdG excretion rate can be used to determine whether a cycle is anovulatory, ovulatory and infertile, or ovulatory and fertile [49]. In the study by Blackwell et al. 2018, follicular growth was found to be indicated by a peak in E1-3G excretion, while ovulation was indicated by a subsequent post-ovulatory rise in PdG. The authors emphasised the potential of measuring these markers as an indication of the nature of infertility, assessing whether intervention (such as ovulation induction with clomiphene) is warranted, and in identifying the most appropriate day for an ultrasound scan or gonadotropin dose adjustment [49].

A number of studies have evaluated the use of urinary assays to monitor induction of ovulation. The first successful induction of ovulation in hypogonadotrophic anovulatory women who achieved pregnancy was reported in 1962. In the referred study, a sequential step-up/step-down regimen was followed, in which cycle monitoring was performed using only urine-based hormonal monitoring [1, 2]. Later, Maclean et al. developed a direct urinary RIA for E1-3G and PdG with the aim of increasing the capacity of an ovulation induction programme in women receiving treatment for infertility [71]. In these women, E1-3G was found to be relatively similar to that of fertile women at various stages of the menstrual cycle, with a decrease in E1-3G towards pre-treatment levels observed in cycles which did not result in pregnancy. The authors concluded that urinary analysis of hormones is a reliable method to monitor indices of ovarian function in patients receiving treatment for ovulation induction [71]. This was supported in a study by Lessing et al., in which hormones were monitored by urinary analysis in conjunction with ultrasound measurement of follicle size, in 31 women undergoing induction of ovulation [58]. Additionally, the RIA developed by Catalan et al. demonstrated a highly significant correlation between serum E2 and urinary E1-3G in normal menstruating women and in those undergoing ovulation induction (r = 0.9209 and r = 0.9229, respectively; both *P* < 0.01) [52].

Rapi et al. also used urinary assays to monitor ovulation and assess correlation with follicular growth (a principal parameter in evidencing a successfully induced cycle) in patients undergoing COS, IVF and embryo transfer. Although results showed that correlation between E1-3G and follicular size presented a large individual variability, the urinary assay was determined to be a reliable method for detecting the optimal hCG administration day during IVF treatment [47]. Similarly, in women undergoing COS with hMG, urinary E1C levels correlated with serum E2 levels, as determined by EIA and RIA, respectively [60].

**Fig. 1:**
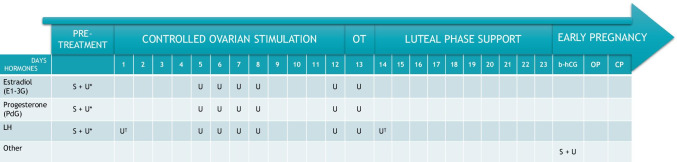
Specialist recommendation for the days on which hormonal monitoring could potentially be performed using urine-based assays (based on the results of 2019 survey). b-hCG; beta human chorionic gonadotrophin (pregnancy test); CP, clinical pregnancy; OP, ongoing pregnancy; OT, ovulation triggering (either with human chorionic gonadotrophin or gonadotrophin-releasing hormone agonist); S, serum-based hormonal assay; U, urine-based hormonal assay. *Additional hormonal assessments performed in the case of ovulation triggering with human chorionic gonadotrophin. †Additional hormonal assessments performed in the case of ovulation triggering with gonadotrophin-releasing hormone agonist

In addition, during the 2019 survey, the specialists (N = 7) were asked to specify the days during the fresh embryo transfer cycle on which hormonal monitoring could potentially be performed using urine-based assays. Based on the recommendation from the specialists, urine-based hormonal testing could potentially be performed between Days 5–8 and 12–13 of COS, with additional urinary hormone assessments proposed on Day 1 and Day 14 of COS in the cases when ovulation triggering is performed with GnRH agonist (Fig. 1).

## Telemedicine and Remote Treatment Monitoring

### Value of Telemedicine

While there is no one definition of telemedicine, the concept involves a wide spectrum of systems for the delivery of health services that substitute the classical means of personal communication with electronic communication [72]. Telemedicine challenges the traditional face-to-face patient‒doctor interaction and has, in certain contexts, been embraced positively by patients and healthcare practitioners (HCPs) specifically for improving access to health services [73, 74]. Telemedicine services have developed significantly in the past decade, and there is fair evidence supporting their effectiveness and efficiency [73].

### Success of Telemedicine and Remote Treatment Monitoring Across a Number of Therapeutic Areas

One of the first therapeutic areas in which telemedicine showed significant promise was multiple sclerosis (MS). Results of a longitudinal study showed that telemedicine by telephone represents an easy and efficient method for monitoring medication use and adherence among individuals with MS [75]. In addition, wearable technologies, to enable remote treatment monitoring, were reported as promising tools for patients with MS, although assessment of their reliability and accuracy is warranted [76]. Other recognised examples of remote monitoring include home-based blood pressure monitoring, remote blood glucose monitoring in patients with diabetes and home-based monitoring of pulmonary function in patients with Duchenne muscular dystrophy [77–79].

### Current Evidence for Telemedicine and Remote Treatment Monitoring in Fertility

The concept of self-operated endovaginal telemonitoring (SOET) was first introduced in 2009, in order to reduce the number of monitoring visits at the clinic [80, 81]. It is a reusable portable sonographic device connected with an FDA approved, CE marked endovaginal probe [82]. A randomized controlled trial of 121 analyzed patients was subsequently conducted, in which patients recorded vaginal sonograms at home and sent recordings using a cloud-based device to the care provider. During this study, conception and ongoing pregnancy rates resulting from SOET-monitored treatment cycles were similar to those from cycles monitored via traditional methods, but with reduced overall costs [83]. Patient-reported outcomes, including feelings of empowerment, discretion, partner involvement and stress, were also more favourable for SOET compared with traditional monitoring [83]. However, it should be noted that the methods used for patient-reported outcomes in this study were not well described and may present a risk of bias, as they were based on mostly author-developed questionnaires completed at post-study interviews by the staff [83]. A later cohort study of 100 attempts at home sonography also showed that the results were similar to those using traditional clinic-based monitoring, and that 90% of patients could avoid clinic visits for sonography [82]. Another small cohort study from Germany reported similar findings [84]. From the patient perspective, home sonography confers many advantages: greater flexibility for patients and their partners, less loss of income to attend appointments during working hours especially for those who live far away from clinics, and a more environmentally friendly approach to treatment due to the reduced travel [85].

Another example of patient experience assessment with home-based monitoring comes from urinary LH testing to predict ovulation. A study by Zaat et al. evaluated patient experience regarding hospital-based monitoring versus home-based monitoring (using a urinary LH test kit to guide the timing of frozen-thawed embryo transfer) in natural and gonadotropin-stimulated cycles [86]. The analysis was performed on a sample of 116 women using home-based monitoring and 116 women using hospital-based monitoring [86]. The method of monitoring was shown to have a significant effect on patient experience in favour of home-based monitoring [87]. Although the questionnaire used to evaluate patient-reported experience measures was developed by the authors and its reliability had not been demonstrated, face-validity was shown for three items of the questionnaire, which were of importance to patients [86].

Additionally, a prospective study was conducted to test the home use of the ClearPlan® fertility monitor, which simultaneously detects LH and E1-3G in early morning urine to delineate three levels of fertility: low, high and peak, the latter resulting from the surge in LH [88]. In this study, the fertility monitor was used by 53 women to predict ovulation in natural cycles and the results were compared with conventional methods, including transvaginal ultrasound and serum hormone measurements. Home-based monitoring of urinary LH and E1-3G was shown to accurately predict a two-day window for ovulation in 91.1% of cycles. The authors concluded that the monitor could potentially be used as a diagnostic aid and for monitoring the treatment of infertility, as the system allows the storage of patients’ data for several months, which can be evaluated retrospectively [88].

### Implications for Future Research in MAR and Possible Applications in other Areas of Reproductive Health

Despite the available evidence on the potential benefits and clinical applications of telemedicine and remote urine-based hormonal testing for fertility treatment, further evidence is needed to validate such methods for clinical use. Firstly, the accuracy and effectiveness of home-based urinary hormonal telemonitoring versus current standard-of-care (clinic-based serum hormonal monitoring and clinic-based ultrasound assessment of ovarian response by measuring number and size of growing follicles) should be assessed in clinical studies as well as in real-world clinical practice. As home-based urinary monitoring is likely to involve fewer clinic visits, studies should also assess if this affects treatment decisions made by the clinician (as compared with more conventional monitoring in the clinic). Although the literature suggests an overall positive patient experience with home-based fertility monitoring, the limitations of the available evidence regarding study design and methodology may impact the interpretation and application of currently available studies. As such, patient-reported outcomes and experience including psychosocial wellbeing should be assessed with psychometrically tested, reliable and validated questionnaires to gather a robust evidence base. Health economic studies of direct and indirect costs would also be beneficial, including ecological, economical and psychological aspects. Finally, policy makers should be encouraged to promote the expansion of telemedicine, especially in light of the COVID-19 pandemic, so that the private health insurance companies and state funding support the migration of health services to the digital platforms.

The ultimate goal of telemedicine in fertility would be to create a modular ecosystem which links clinic staff to patients at home, enabling patients to perform remote treatment monitoring with the guidance of their physician through telecounselling (or other digital channels), without diminishing the positive patient-reported aspects associated with frequent HCP communication and support. In this way, we establish patient-friendly solutions for cycle monitoring, rapid and convenient communication between patient and physician, while reducing the need for blood withdrawal, multiple clinics visits, long waiting times and other potentially burdensome aspects of cycle monitoring [89]. Furthermore, by using telemedicine in conjunction with urinary monitoring, some of the disadvantages of this method previously observed in clinics are less common; there is less interference to the life of the patient as only one sample of morning urine is collected and analysed by the patient at home, without the need to accumulate, store or transport multiple urine samples. Results of remote urine-based hormone assays could be sent as encrypted data through the internet to the clinic. In the clinic, specialized personnel would receive, store, analyze and interpret the images/parameters via novel technology, leading to results-based interventions, i.e. dosage adjustment or next-step decisions. Any remote monitoring device provided to fertility patients should be convenient, user friendly, time efficient, easy to transport and maintain, robust and reliable in producing accurate results, in addition to providing secure data storage and transmission.

Hormone monitoring is also used during early pregnancy to assess the developing fetus, offering the opportunity to intervene and prevent miscarriage [1, 3, 20, 90, 91], and could be of benefit to other areas of reproductive health. A pilot study assessed the use of a multi-level pregnancy test based on self-monitoring of urinary hCG trends after assisted reproduction [92]. The results found that 73% of women reported being ‘satisfied’ or ‘very satisfied’ with the home tests and 96.6% found it ‘easy’ or ‘very easy’ to use, and home test results were generally consistent with the results of clinic-based serum hCG testing [92]. Urinary hormone profiling has shown promise for studying corpus luteum deficiency, which may occur during early pregnancy as a result of insufficient progesterone levels. Low luteal phase serum P4 has been used as a diagnostic tool to detect this; however, the rapid fluctuations in circulating P4 due to its pulsatile release from the corpus luteum make serum concentrations unreliable [93]. In contrast, a further study by Magini et al. of ovarian function in a number of pathological conditions, luteal insufficiency was detected in 9/15 women affected by habitual miscarriage and was associated with a significantly higher ratio of E1-3G to PdG throughout the luteal phase [94]. This suggests that urinary measurements may prove beneficial over serum assessments, as the former are not subject to rapid fluctuations due to the pulsatile release and may, therefore, be more reliable. Further studies suggest that urinary hormonal metabolite assessments (namely, PdG, LH and E1-3G) could help in the diagnosis of luteal deficiency and to treat identified abnormalities in a properly timed, restorative manner [64, 95].

## Conclusions

Cycle monitoring via ultrasound and serum-based hormonal assays during MAR is currently considered standard of care, as it provides information on ovarian response and assists in optimizing treatment outcomes and avoiding complications. However, blood tests may cause inconvenience to patients due to repeated venepuncture and the need for frequent clinic appointments. Remote hormonal monitoring based on urinary assessment of reproductive hormones could be part of a novel digital health solution that includes remote ultrasound and telecounselling to link clinics and patients at home. Especially during the unprecedented times of the current and post-COVID-19 eras, the prospect of a validated remote urinary monitoring system could add value and support decision making during MAR treatment, with the potential to significantly improve overall patient experience.

## Supplementary Information

Below is the link to the electronic supplementary material.

Supplementary file1 (DOCX 23 KB)

## Data Availability

All data analysed during this study are included in this published article and its supplementary information files. Any requests for data by qualified scientific and medical researchers for legitimate research purposes will be subject to Merck KGaA’s Data Sharing Policy. All requests should be submitted in writing to Merck KGaA’s data sharing portal https://www.merckgroup.com/en/research/our-approach-to-research-and-development/healthcare/clinical-trials/commitment-responsible-data-sharing.html. When Merck KGaA has a co-research, co-development, or co-marketing or co-promotion agreement, or when the product has been out-licensed, the responsibility for disclosure might be dependent on the agreement between parties. Under these circumstances, Merck KGaA will endeavour to gain agreement to share data in response to requests.
