# Supplementary material for: Self-Monitoring of Urinary Hormones in Combination with Telemedicine — a Timely Review and Opinion Piece in Medically Assisted Reproduction
Source: Reprod Sci. 2021 Nov 15;29(11):3147–60. doi: 10.1007/s43032-021-00754-5 (PMC8592080; doi:10.1007/s43032-021-00754-5)
Supplement: Supplementary file 1 — Supplementary file1 (DOCX 23 KB) [file 43032_2021_754_MOESM1_ESM.docx]

**Supplementary Table 1: Survey (2019) on hormonal monitoring by fertility specialists**

| **Respondents** | |
| --- | --- |
| N | 7 |
| Specialization | Obstetrician-gynecologist with sub-specialty in fertility or reproductive medicine (n=7) |
| Work experience | > 10 years |
| Country of origin | Belgium (n=1), Germany (n=1), Israel (n=1), Spain (n=3), Australia (n=1) |
| **Survey** | |
| Format | Multiple choice and short answer text |
| Response rate | 100% |
| **Multiple-choice questions (Yes/No)** | |
| Do you routinely check serum hormonal levels [estradiol (E2), luteinizing hormone (LH), progesterone (P4)] during assisted reproductive technology (ART) treatments, to help you monitor controlled ovarian stimulation (COS) or luteal phase support (LPS)? | Y: 7/7 N: 0/7 |
| Do you think hormonal tests during COS and LPS on a regular basis can improve pregnancy and safety outcomes? | Y: 7/7 N: 0/7 |
| Do you think serum E2 levels are an indicative marker of COS when you adjust dose of gonadotropins (Gn)? | Y: 7/7  N: 0/7 |
| Do you see serum E2 levels as an indicator of ovarian hyperstimulation syndrome (OHSS)? | Y: 5/7  N: 2/7 |
| Do you think the peak of E2 levels is an indicator of timing ovulation triggering? | Y: 5/7 N: 2/7 |
| Would you check E2 and/or P4 levels to determine the embryo transfer strategy (fresh vs. frozen embryo transfer)? | E2 levels: 0/7 P4 levels: 0/7 Both of them: 7/7 Neither of them: 0/7 |
| Do you think it is worth monitoring LH and FSH levels after GnRH-agonist triggering? | Y: 5/7 N: 2/7 |
| Do you think hormonal tests during COS and LPS on a regular basis can improve pregnancy and safety outcomes? | Y: 7/7 N: 0/7 |
| **Open-ended questions** | |
| What are the benefits of using serum based hormonal assays during fertility treatment? | For clinician:   1. Absolute readings 2. Exact decisions 3. Easier to avoid OHSS risk 4. To measure E2 and P4 levels in time   For patients:   1. Better compliance 2. Patient friendly 3. Reduced time to pregnancy 4. Guidance on cycle cancellation strategy 5. Guidance on LH supplementation strategy |
| What do you think are the key barriers of using serum-based hormonal assays in fertility treatment? | For clinician:   1. Validity of results 2. Time to get the results 3. New methods are sometimes difficult in convincing clinicians   For patients:   1. Travel 2. Waiting times 3. Discomfort |

**Supplementary Table 2: Survey (2020) on hormonal monitoring by fertility specialists**

| **Respondents** | | | | | | |
| --- | --- | --- | --- | --- | --- | --- |
| N | 17 | | | | | |
| Specialization | Obstetrician-gynecologist with sub-specialty in fertility (n=12), reproductive endocrinologist (n=2), other fertility specialist (n=3) | | | | | |
| Work experience | Under 10 years (n=6), 11–20 years (n=3), 21–30 years (n=6), 31–40 years (n=1) | | | | | |
| Country of origin | Germany (n=4), Spain (n=4), Italy (n=3), France (n=2), India (n=2), Brazil (n=1), Sweden (n=1) | | | | | |
| **Survey** | | | | | | |
| **Format** | Multiple choice and short answer text | | | | | |
| Response rate | 100% | | | | | |
| **Multiple-choice questions (Yes/No)** | | | | | | |
| Do you routinely check serum hormonal levels [e.g. estradiol (E2), luteinizing hormone (LH), progesterone (P4)] during assisted reproductive technology (ART) treatments? | Y: 15/17  N: 2/17 | | | | | |
| **Multiple-choice questions (6-point scale)** | **1 = not well at all** | **2** | **3** | **4** | **5** | **6 = Very well** |
| How well do you think serum E2 levels serve as an indicative marker of Controlled Ovarian Stimulation when you adjust dose of gonadotropins (Gn)? | 1 | 0 | 3 | 4 | 6 | 3 |
| How well do you think serum E2 levels serve as an indicative marker of ovarian hyperstimulation syndrome (OHSS)? | 0 | 2 | 0 | 3 | 8 | 4 |
| How well do you think E2 and/or LH levels can indicate the starting day of GnRH-antagonist administration in flexible protocol?  E2 level alone  LH level alone  E2 and LH in combination | 4  6  3 | 3  7  3 | 2  3  3 | 5  0  4 | 2  1  2 | 1  0  2 |
| How well do you think the peak of E2 levels can indicate the timing of ovulation triggering? | 2 | 4 | 3 | 3 | 2 | 3 |
| **Multiple-choice questions (6-point scale)** | **1 = not at all** | **2** | **3** | **4** | **5** | **6 = very much** |
| To what extent do you think hormonal tests during Controlled Ovarian Stimulation and Luteal Phase Support on a regular basis can improve pregnancy and safety outcomes? | 0 | 2 | 1 | 5 | 6 | 3 |
| **Multiple-choice questions (6-point scale)** | **1 = not important at all** | **2** | **3** | **4** | **5** | **6 = very important** |
| To what extent do you think it is important to monitor LH and FSH levels after GnRH-agonist triggering? | 7 | 4 | 3 | 0 | 1 | 2 |
| To what extend do you think hormonal tests during Controlled Ovarian Stimulation and Luteal Phase Support on a regular basis can improve pregnancy and safety outcomes? | 0 | 2 | 1 | 5 | 6 | 3 |
| **Open-ended questions** | | | | | | |
| Which hormones do you measure? | E2: 15/15  P4: 12/15  LH: 10/15  Other: 3/15 | | | | | |
| What role do hormone test play in regard to Controlled Ovarian Stimulation and Luteal Phase Support in your practice? What can they deliver and what are their shortcomings? | Advantages:   - Help to make more informed treatment decisions   Disadvantages:   - Logistics of the patient having to undergo blood test ideally for each step of monitoring is a nuisance - Duration until results are back is too long - Worries about measuring errors: inter-cycle vs. intra-cycle; between different labs; due to circadian rhythm (esp. for progesterone) - Need for a blood sample | | | | | |
| Apart from hormone measures what other diagnostics do you carry out? For what reason? | Ultrasound: 17/17 | | | | | |
